# The Interdependency of the Morphological Variations of the Planktonic Foraminiferal Species *Globigerina bulloides* in Surface Sediments on the Environmental Parameters of the Southwestern Indian Ocean

**DOI:** 10.1155/2014/621479

**Published:** 2014-10-29

**Authors:** Abhijit Mazumder, Neloy Khare, Pawan Govil

**Affiliations:** ^1^Birbal Sahni Institute of Palaeobotany, 53 University Road, Lucknow 226 007, India; ^2^Ministry of Earth Sciences, Prithvi Bhawan, Near India Habitat Centre, Lodhi Road, New Delhi 110 003, India

## Abstract

18 surface sediment samples collected from a north-south transect along the Indian Ocean have been analyzed for planktonic Foraminifera content. Among the other planktonic foraminiferal faunas, *Globigerina bulloides* was present substantially in all samples. Census data of *G. bulloides* were measured for different parameters (average size, mean proloculus size, coiling direction, and number of chambers) and a Q-mode cluster analysis was applied on these data. Samples were segregated into two homogeneous clusters, each reflecting particular environmental conditions. Two clusters are as follows: (1) Cluster A, comprised of 6 samples and characterized by the highest range of foraminiferal and ecological parameters, except sea surface temperature and salinity which shows the lowest range, and (2) Cluster B, comprised of 12 samples and characterized by the lowest range of foraminiferal parameters and ecological parameters, except sea surface temperature and salinity which shows the highest range. The study suggests that the ecological parameters are the governing factors for the morphological characteristics of planktonic foraminiferal species *G. bulloides*.

## 1. Introduction

Morphological variations of planktic Foraminifera have been extensively used to decipher paleoclimatic, paleoenvironmental, and paleoecological reconstructions [1–3].

*Globigerina bulloides* d'Orbigny, a spinose planktic foraminifer, is substantially present in temperate to subpolar water masses and is also characteristic of upwelling areas in lower latitudes [4–11]. In these upwelling regions,* G. bulloides* contributes maximum foraminiferal flux to the ocean floor [12, 13] and therefore provides important geochemical information for paleoceanographic studies [14–17].

Although earlier workers [18–21] discussed the general distribution of* G. bulloides* in Indian Ocean surface waters and sediments along with its relation to the change of ecological parameters, no attempt was made to study the morphological variations of this planktic foraminiferal species along a north-south transect in the Indian Ocean region in connection with the ecological parameters.

In the present work, the results of a biometric study of the latitudinal variation in test size, proloculus size, number of chambers, and coiling direction of* G. bulloides* from the surface sediments of a north-south transect of the Indian Ocean were analyzed. The major objective of this study is to link the patterns of morphological variation with the changes in physicochemical properties of the surface water in order to comprehend the ecological control on morphological characteristics in* G. bulloides* in the modern marine environment.

## 2. Study Area

**Figure 1:**
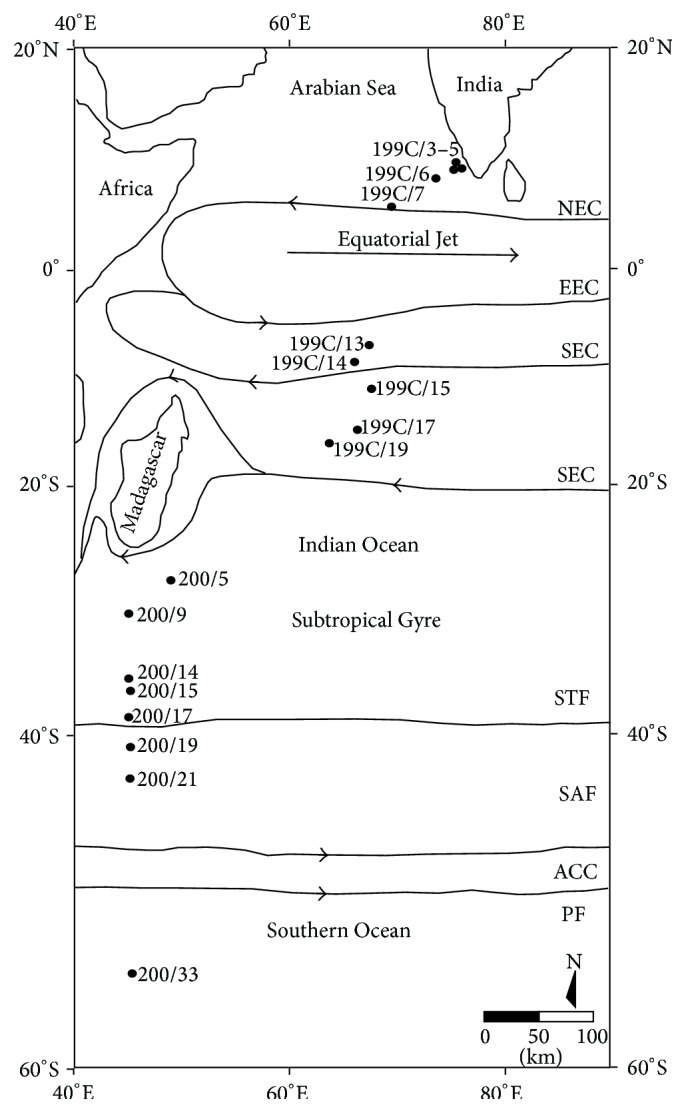
Location of sampling stations along a north-south transect in the southwestern Indian Ocean (NEC: North Equatorial Current; EEC: Eastward Equatorial Current; SEC: South Equatorial Current; STF: Subtropical Front; SAF: Subantarctic Front; ACC: Antarctic Circumpolar Current; PF: Polar Front).

The study area falls within the southwestern Indian Ocean basin. Previously, Ichiye [22], Wyrtki [23], and Gordon [24] gave a detailed account of the physical oceanography of the southern Indian Ocean. Two critical water-mass boundaries are present in this area: the Antarctic Polar Front, which separates Antarctic water mass from Subantarctic water mass, and the Subtropical Convergence, which separates Subantarctic from subtropical water masses (Figure 1). The Antarctic Convergence exhibits the sharpest water-mass boundary in the southern Indian Ocean waters in terms of the change in temperature and salinity whereas the gradients at the Subtropical Convergence (38°S–40°S) are less steep.

During the pilot expedition to the Southern Ocean (PESO) aboard the Oceanic Research Vessel* Sagar Kanya* (199C and 200th cruises), a total of 18 surface sediment samples (0-1 cm) (including core top and grab samples) were collected along a north-south transect between 9.50°N to 45°S latitude and 80°E to 40°E longitude, covering tropical, subtropical, and Subantarctic waters in the southwestern Indian Ocean to undertake planktonic foraminiferal analysis (Figure 1, Table 1).

## 3. Hydrologic Setting

The sampling stations are characterized by several distinct water regions spreading over latitudinal segments: tropical, subtropical, transitional, and Subantarctic. They are divided into these waterbodies depending upon several zoogeographic provinces which are mainly influenced by ecological and climatological parameters [4, 25]. Southern Ocean can be divided into three prominent zones based on the water dynamics: the Western Boundary Current (WBC) zone (between 35° and 45°S), the Antarctic Circumpolar Current (ACC) zone (between 45° and 60°S), and the Seasonal Sea Ice (SSI) zone (between 60° and 75°S). The WBC zone comprises of mainly three currents at western boundary; they are the Agulhas Current, the Brazil/Malvinas Current, and the East Australia Current. Hydrographic conditions in Southern Ocean (SO) are mainly controlled by an eastward flowing Antarctic Circumpolar Current (ACC) [26].

The western part of the southern Indian Ocean acquires heat from the warm western boundary current [27]. The available data on the hydrological fronts and freshwater input along 62°E and 30°E sections [28] highlighted that the areas west of the Crozet Plateau and east of the Kerguelen-Amsterdam passage are two vital regions where the fronts diverse and converse. Agulhas Return Front (ARF), Southern Subtropical Front (SSTF), and Northern Subantarctic Front (NSAF) were designated as the combined front between 40°15′S and 43°S suggesting that the combined isotherms exhibit temperature variation from 19°C to 10°C and the combined isohalines exhibit a drop in salinity from 35.54 to 34.11 psu across ~3° latitude [26].

The Southern Subantarctic Front is situated between 47° and 48°S (between 6 and 7°C isotherms). The Polar Front (PF1) is present between 49° and 50°S (isotherms varied from 5 to 4°C). Southern Polar Front (PF2) is present between 52° and 54°S (temperature range 3-2°C). Antarctica Intermediate Water (AAIW) flows at ~1150 and ~1200 m water depth and the characteristics of this water mass were recorded as temperature of ~4.4°C, minimum salinity of ~34.42 psu, and density of ~27.24 kg m^−3^ in the northern front of subtropical zone [29].

Circumpolar Deep Water is characterized by different features, such as temperature of ~2°C, salinity of ~34.77 psu, and density of ~27.8 kg m^−3^. It flows at the water depth between 2000 and 3800 m north of 45°S and rises sharply to shallower depths south of the frontal zone. North Atlantic Deep Water (NADW) with higher salinities (~34.8 psu) transported from the South Atlantic to the southwestern corner of the Indian Ocean and Madagascar ridge blocks NADW to reach east of ~45°E [30]. Below the CDW, temperature and salinity decrease due to the influence of Antarctica Bottom Water (AABW). AABW is recorded between 49° and 56°S at the depth of 4100 to 4700 m with a temperature of ~−0.165 to −0.62°C, salinity of ~34.67 to 34.65 psu, and density of ~27.85 to 27.86 kg m^−3^ [26]. The Antarctic Circumpolar Current (ACC) reaches the ocean floor to mix with the North Atlantic Deep Water (NADW) as well as deep waters from the Indian Ocean and the Pacific Ocean. Thus, the mixture of these deep waters along with the Circumpolar Deep Waters (CDW) spreads to other oceans basins.

AABW and CDW enter the Indian Ocean in the west around Madagascar and East Africa and in the east along the Ninety East Ridge [31]. A very strong and deep overturning cell about 1800 m below near 32°S carries layers of warm near-surface water and cold deep water in opposite directions [30]. The Southern Ocean has also a unique role in the global scale overturning circulation caused by the circumpolar connection in the Southern Ocean. Water found at intermediate and abyssal depths at low latitudes rises towards the surface in the Southern Ocean. Deep water that upwells closer to Antarctica loses its heat after coming in contact with the cold air blowing off the continent and its salinity is eventually increased by brine released during sea ice formation. The dense water formed in this process gradually sinks near the continental margin of Antarctica and takes a return path to the north in deep currents flowing along the sea floor.

## 4. Methods

All the sediment samples were processed as per standard procedures. An appropriate amount of sediment (~5 gm) from each sample was dried overnight at 45°C. Dried sediment samples were soaked in water and subsequently treated with sodium hexametaphosphate in order to dissociate clay lumps. The treated sediments were sieved over 63 *μ*m sieve and dried and transferred to plastic vials. While processing the sediment samples, utmost care was taken to prevent any possible breaking of the foraminiferal test, by using extremely low water pressure for removing the finer (>63 *μ*m) fraction. The >63 *μ*m fraction was dry sieved and, from the >125 *μ*m fraction, an aliquot was taken by quartering and coning, to pick a minimum of 40 specimens of the planktic foraminiferal species* G. bulloides*.

After the picking procedure, morphological features such as coiling direction, shell size, number of chambers, and proloculus size were observed and measured under a stereo zoom microscope using a scale with divisions of 14 *μ*m. The test size was measured along the last chamber to its diagonally opposite chamber through the apex; coiling direction was observed from dorsal side of the test, while the number of chambers was counted starting from the proloculus chamber to the last chamber observed on dorsal side of the test. The average values of these four parameters of all 40 individual specimens from each location were presented as mean values for each location (Table 1).

**Figure 2:**
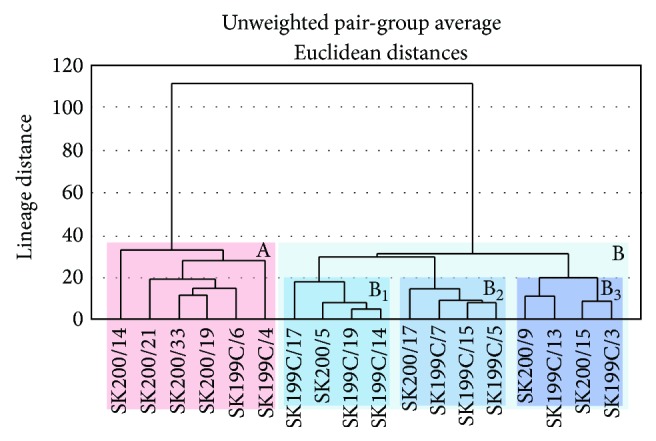
Results of the cluster analysis of the four morphological parameters (coiling direction, mean proloculus size (MPS), average test size, and number of chambers) analysed from the planktonic foraminiferal species* Globigerina bulloides*.

The data obtained was subjected to Q-mode cluster analysis. The analysis was performed using the unweighted pair group averaging method (Figure 2). The morphological parameters were chosen as variables to increase the precision of the analysis. The results of cluster analysis are plotted in the form of a two-dimensional hierarchy dendrogram wherein locations are presented along the *x*-axis while similarity level is plotted on *y*-axis (Figure 2). This dataset was compared with the modern hydrological settings (sea surface temperature, sea surface salinity, nutrients, and dissolved oxygen; mean values of 0 m, 50 m, 100 m, 150 m, and 200 m, considered the average value of 0–200 m water column) along the north-south transect in the present study area. This hydrological data was retrieved from World Ocean Atlas [32–35]. Moreover, the clusterwise comparative chart of morphological parameters and ecological parameters is tabulated to show the correlation between these two (Table 2).

## 5. Results

The Q-mode cluster analysis classified the samples into two homogeneous clusters (A and B) under the linkage distance 40 (Figure 2). Cluster B was in turn subdivided into subclusters B_1_, B_2_, and B_3_ under the linkage distance 20. Each cluster and subcluster is characterized by a particular association of ecological parameters. The following are the relation of clusters and subclusters with ecological parameters.

Cluster A comprises a total of 6 samples, of which two fall between the latitudes 9.4051°N and 8.1333°N (tropical zone) with the remaining samples within the latitudes 36.12°S and 45°S (towards the subpolar zone). This cluster is characterized by the highest range of all morphological characteristics: average test size (range 336.82–383.19 *μ*m; average 357.33 *μ*m), mean proloculus size (range 11.89–16.73 *μ*m; average 15.19 *μ*m), number of chambers (range 9.57–10.64; average 10.23), and dextrality (range 54.54–76.19%; average 70.31%). Sea surface temperature (SST) and sea surface salinity (SSS) show the lowest range of values between 1.14–24.23°C (average 14.61) and 34.06–35.50 psu (average 34.77 psu), respectively. On the other hand, the nitrate, phosphate, and dissolved oxygen contents of the water show the highest values. The nitrate content ranges from 1.165 *μ*mol to 7.81 *μ*mol with an average of 4.14 *μ*mol, while the phosphate content shows a range of 1.774 *μ*mol to 8.423 *μ*mol with an average of 4.07 *μ*mol, and total nutrients show a range of 4.202 *μ*mol to 11.547 *μ*mol with an average of 8.21 *μ*mol. The dissolved oxygen ranges from 3.886 mg/L to 7.733 mg/L with an average 5.34 mg/L (Table 2).

Cluster B (12 samples) comprises of samples from a wider range of the study area (9.5040°N to 39.03°S latitudes), which mainly falls within the tropical to subtropical zones. This cluster is characterized by the lowest range of all morphological characteristics: average test size (range 225.35–271.00 *μ*m; average 247.65 *μ*m), mean proloculus size (range 7.07–9.40 *μ*m; average 8.34 *μ*m), number of chambers (range 9.38–11.10; average 10.21), and dextral coiling (range 50.00–88.88%; average 67.32%). Two ecological parameters, namely, SST (range 15.63–23.85°C; average 20.55°C) and SSS (range 33.53–35.50 psu; average 35.03 psu), show the highest ranges. The nutrients and dissolved oxygen showed the lowest ranges. The total nutrient values varied between 1.851 *μ*mol and 11.578 *μ*mol (average 4.28 *μ*mol), while the dissolved oxygen ranged from 4.431 mg/L to 5.612 mg/L (average 4.82 mg/L). The nitrate values ranged from 0.3 *μ*mol to 9.105 *μ*mol (average 1.89 *μ*mol) while phosphate values varied from 1.421 *μ*mol to 5.95 *μ*mol (average 2.39 *μ*mol). Cluster B is further subdivided into three subclusters, namely, B_1_, B_2_, and B_3_ (Table 2).

Subcluster B_1_ consists of four samples, located within the latitudes of 9.179°S and 28.32°S. This subcluster is characterized by the lowest range of average test size (range 225.35–245.05 *μ*m; average 238.03 *μ*m) and dextrality (range 50.00–60.00%; average 54.29%). On the other hand, it shows the highest range of number of chambers (range 10.05–11.10; average 10.54) and moderate range of mean proloculus size (range 7.96–8.64 *μ*m; average 8.23 *μ*m). In the case of ecological parameters, this cluster shows a lower range of values with the exception of SST and SSS. SST shows the highest values ranging between 19.76 and 22.76°C with an average of 21.27°C. In contrast, SSS ranges moderately within the cluster (range 34.92–35.42 psu; average 35.08 psu). The values of nitrate and phosphate varied from 0.38 to 0.7 *μ*mol (average 0.48 *μ*mol) and from 1.421 to 2.245 *μ*mol (average 1.68 *μ*mol), respectively, which collectively ranged from 1.851 to 2.945 *μ*mol (average 2.17 *μ*mol). Dissolved oxygen ranged from 4.543 mg/L to 4.936 mg/L, with an average of 4.71 mg/L.

Subcluster B_2_, represented by four samples, is distributed between latitudes 5.5121°N and 39.03°S. This subcluster is characterized by the lowest range of mean proloculus size (range 7.07–9.15 *μ*m; average 7.78 *μ*m), number of chambers (range 9.38–10.11; average 9.73), and the highest range of dextrality (range 75.00–88.88%; average 82.85%). On the other hand, it shows a moderate range of average test size (range 58.06–95.65%; average 82.00%) within Cluster B. In the case of ecological parameters, the SSS shows the lowest range (33.53–35.44 psu) with an average of 34.77 psu. SST and dissolved oxygen show medium ranges (15.63–23.85°C, average 21.11°C and 4.431–5.612 mg/L, average 4.87 mg/L, resp.). In the case of nutrients, nitrate shows a medium value (range 0.3–1.07 *μ*mol, average 0.58 *μ*mol), while phosphate shows the highest value (range 1.568–5.95 *μ*mol; average 3.10 *μ*mol) within Cluster B; collectively, the nutrient values range from 1.938 *μ*mol to 7.02 *μ*mol with an average of 3.68 *μ*mol, which shows a medium value within Cluster B.

Subcluster B_3_ consists of four samples, located within the latitudes of 9.5045°N and 37°S. This subcluster is characterized by the highest range of average test size (range 259.50–271.00 *μ*m; average 264.6 *μ*m) and mean proloculus size (range 8.56–9.40 *μ*m; average 9.02 *μ*m) within Cluster B. On the other hand, it shows moderate range of number of chambers (range 9.79–10.71; average 10.36) and dextrality (range 53.84–75.00%; average 64.83%) within Cluster B. In the case of ecological parameters, this cluster shows the lowest range of SST (16.81–22.08°C; average 19.27°C) and highest range of SSS (34.94–35.50 psu; average 35.23) within Cluster B. The value of nitrate varies from 0.3 to 9.105 *μ*mol (average 4.29 *μ*mol), which is highest within Cluster B. Though phosphate shows a moderate range (1.687–4.271 *μ*mol; average 2.56 *μ*mol), collective nutrients show the highest range of values (1.987–11.578 *μ*mol; average 6.85 *μ*mol) within Cluster B. Dissolved oxygen shows a range of 4.495 mg/L to 5.423 mg/L, with an average of 4.90 mg/L, which is highest within Cluster B.

The correlation coefficient between the morphological variations (average test size, proloculus size, number of chambers, and dextrality) and the ecological parameters, namely, SST, SSS, nitrate content, phosphate content, total nutrient (nitrate + phosphate), and dissolved oxygen, was performed (Tables 3(a) and 3(b)). Some correlation was found moderately correlatable, namely, test size-SST, test size-nitrate (hence total nutrient), test size-dissolved oxygen, number of chambers-dissolved oxygen, proloculus size-SST, proloculus size-nitrate (hence total nutrient), and proloculus size-dissolved oxygen.

## 6. Discussion and Conclusion

The foraminiferal data have been subjected so far to statistical analysis, namely, cluster analysis in different geographical regions and for various purposes [36–46].

*G. bulloides* is abundant in high southern latitude water masses and is at its peak in high northern latitudes, low latitude upwelling regions, and nutrient-rich environments [47–50]. In this study, cluster analysis on the morphological characteristics of* G. bulloides* is used to differentiate different water masses depending more on ecological parameters than on latitudinal gradients. In general, two major clusters (Clusters A and B) show that morphological characteristics are directly correlated to nutrients and dissolved oxygen of the ambient water mass and inversely correlated to sea surface temperature and sea surface salinity. From the combined results of all subclusters, it was observed that average test size depends directly on nitrate values and dissolved oxygen content of the ambient environment and is inversely related to the temperature. The average size of* G. bulloides* in Cluster A (357.33 *μ*m) is greater than that in Cluster B (247.65 *μ*m). Comparing the nitrate value, dissolved oxygen content, and temperature, we found that the average nitrate value and dissolved oxygen value of surface water in Cluster A (4.14 psu and 5.34 mg/L, resp.) are much higher than those in Cluster B (1.89 psu and 4.82 mg/L, resp.). In case of the three subclusters of Cluster B, the same trend was also observed (Table 1). Bé et al. [51] and Hecht [52] pioneered the study of the ecological influence on the adult test size of planktic foraminifers. In the North Atlantic region, “environmental optima” were defined for temperature and salinities for many planktonic species including* G. bulloides* [52]. Largest sizes of the same species were reported in the Indian Ocean at temperatures around 6-7°C [53, 54]. A positive correlation between general size and the frequencies of* G. bulloides* suggests that optimum growth occurs in areas of optimum environmental conditions rather than in more marginal environments where delayed reproduction (and hence greater size) might have occurred [53]. The upwelling assemblage, predominated by the species* G. bulloides*, has an affinity to high nutrients, because fertility is the defining characteristic for these assemblages [55].

Mean proloculus size (MPS) increases with the increase in SSS. In all subclusters under Cluster B, the average MPS shows a direct relation with the average SSS. Moreover, in our study, it is recorded that an increase in phosphate leads to a rise in dextrality and number of chambers. In Cluster A, the average dextrality and number of chambers show higher values (70.21% and 10.23, resp.) than those in Cluster B (67.32% and 10.21, resp.), which shows a direct relationship with the higher average phosphate value in Cluster A (4.07 psu) and lower value in Cluster B (2.39 psu). The dextrality is also showing an inverse dependency on the average SST. Cluster A shows higher average dextrality (70.31%) with lower average SST (14.61°C), whereas Cluster B shows lower average dextrality (67.32%) with higher average SST (20.55°C). An association between surface-water temperatures and coiling direction in living* G. bulloides* was reported from the southwestern Atlantic Ocean [56]. Malmgren and Kennett [53] observed a distinct relationship between the average surface-water temperature and coiling direction of* G. bulloides*. However, in both Antarctic Ocean and Indian Ocean, the surface-water temperature and the percentage of sinistral specimens are significantly negatively correlated.

Morphologically defined species of marine plankton often harbor a considerable level of cryptic diversity [57]. The results of present study are purely based on the cluster analyses of the morphological features of planktic species* Globigerina bulloides* on the assumption that each morphospecies of planktonic Foraminifera represents a genetically continuous species with a unique habitat. However, the possibility of having cryptic species in the species level population in the study material may not be ruled out completely. No doubt hidden genetic diversity among modern planktonic Foraminifera has significant repercussions on paleoproxies derived from their fossil shells. Nevertheless, Kucera and Darling [58] have compiled the genetic diversity and found 33 cryptic genetic types in 9 out of the 22 sequenced morphospecies of modern planktonic Foraminifera, implying that the total number of cryptic genetic types per morphospecies is not large and that most genetic types show a nonrandom pattern of distribution in the oceans [58]. Furthermore, Morard et al. [59] also pointed out that the cryptic genetic species of planktonic Foraminifera often exhibit narrower biogeographic distributions and ecological preferences than the respective morphospecies. In theory, it should therefore be possible to improve the resolution of the paleoceanographic reconstructions based on sediment assemblages of these species. Since many morphospecies show cosmopolitan distribution, an understanding of biogeographic and evolutionary processes at the level of genetic diversity requires global sampling [57]. Such an approach is beyond the scope of the present study.

Though the inferences drawn in the present study based on Q-mode cluster analysis clearly establish a correspondence between the ecological parameters of the ambient water masses and the morphological variables of the planktic foraminiferal species* G. bulloides*, more transects covering a wide geographical region need to be covered for arriving at a clearer conclusion.

## Figures and Tables

**Table 1:** Average values (mean as open values and range in parenthesis) of the morphological parameters of *G. bulloides *for each sampling location.

Sample number	Latitude	Test size (*µ*m) [range]	MPS (*µ*m) [range]	Number of chambers [range]	Dextral %
SK199C/3	9.5045	268.00 [192–348]	8.56 [6–12]	10.33 [9–12]	73.33
SK199C/4	9.4051	336.82 [260–442]	15.72 [10.4–6]	10.64 [9–12]	54.54
SK199C/5	8.9917	241.50 [180–324]	9.15 [6–18]	9.63 [8–12]	87.50
SK199C/6	8.1333	347.90 [260–429]	14.55 [13–26]	10.48 [8–13]	66.66
SK199C/7	5.5121	233.33 [204–276]	7.07 [6–12]	10.11 [9–14]	88.88
SK199C/13	−7.3648	260.14 [216–312]	8.99 [6–18]	10.71 [9–13]	57.14
SK199C/14	−9.1790	243.20 [204–312]	8.64 [6–18]	10.40 [8–13]	60.00
SK199C/15	−11.4243	238.00 [156–325]	7.47 [6–13]	9.80 [8–12]	80.00
SK199C/17	−15.2785	225.35 [156–336]	7.96 [6–13]	11.10 [8–14]	50.00
SK199C/19	−16.2677	245.05 [182–312]	8.13 [6.5–13]	10.05 [8–13]	55.00
SK200/5	−28.3215	238.52 [156–312]	8.20 [6.5–13]	10.61 [9–12]	52.17
SK200/9	−30.9142	271.00 [195–442]	9.40 [6.5–26]	10.62 [8–12]	53.84
SK200/14	−36.1217	383.19 [257.1–485.7]	11.89 [7.1–21.4]	10.12 [8–12]	73.53
SK200/15	−37.0000	259.50 [180–348]	9.15 [6–18]	9.79 [8–13]	75.00
SK200/17	−39.0285	248.25 [192–336]	7.43 [6–18]	9.38 [8–10]	75.00
SK200/19	−40.9813	363.05 [192–520]	16.55 [6–26]	10.24 [8–12]	76.19
SK200/21	−43.1500	352.73 [273–416]	16.73 [10.4–26]	10.33 [8–13]	86.66
SK200/33	−55.0065	360.29 [260–520]	15.69 [20.4–26]	9.57 [8–12]	64.28

**Table 2:** Comparative morphological and ecological data for each cluster/subcluster for *G. bulloides*.

Clusters	Subclusters	Morphological parameters				Ecological parameters				
		Avg. test size (*µ*m)	Avg. MPS (*µ*m)	Avg. number of chambers	Avg. dextrality (%)	Avg. SST (°C)	Avg. SSS (‰)	Avg. nitrate (psu)	Avg. phosphate (psu)	Avg. dissolved O_2_ (mg/L)
A		357.33	15.19	10.23	70.31	14.61	34.77	4.14	4.07	5.34
B		247.65	8.34	10.21	67.32	20.55	35.03	1.89	2.39	4.82
	B_1_	238.03	8.23	10.54	54.29	21.27	35.08	0.48	1.68	4.71
	B_2_	240.27	7.78	9.73	82.85	21.11	34.77	0.58	3.10	4.87
	B_3_	264.66	9.02	10.36	64.83	19.27	35.23	4.29	2.56	4.90

**(a):** 

	SST	SSS	Nitrate	Phosphate	Total nutrient	Dissolved oxygen
Test size	*y* = (−0.058)*x* + 35.30	*y* = (−0.002)*x* + 35.41	*y* = 0.031*x* − 5.988	*y* = 0.009*x* − 0.032	*y* = 0.040*x* − 5.944	*y* = 0.009*x* + 2.499
Number of chambers	*y* = 4.773*x* − 3019	*y* = 0.364*x* + 31.22	*y* = 0.232*x* + 0.744	*y* = (−1.562)*x* + 18.68	*y* = (−1.317)*x* + 19.32	*y* = (−0.977)*x* + 15.18
Proloculus size	*y* = (−0.926)*x* + 28.41	*y* = (−0.056)*x* + 35.54	*y* = 0.389*x* − 1.096	*y* = 0.106*x* + 1.52	*y* = 0.492*x* + 0.493	*y* = 0.147*x* + 3.58
Dextrality	*y* = (−0.066)*x* + 23.11	*y* = (−0.013)*x* + 35.87	*y* = 0.026*x* + 1.331	*y* = 0.043*x* − 0.259	*y* = 0.072*x* + 0.969	*y* = 0.018*x* + 3.935

**(b):** 

	SST	SSS	Nitrate	Phosphate	Total nutrient	Dissolved oxygen
Test size	**0.289**	0.029	**0.329**	0.073	**0.352**	**0.322**
Number of chambers	0.13	0.097	0.001	0.129	0.023	**0.226**
Proloculus size	**0.292**	0.14	**0.205**	0.039	**0.209**	**0.341**
Dextrality	0.02	0.109	0.011	0.078	0.054	0.062
